# Executive and Language Control in the Multilingual Brain

**DOI:** 10.1155/2014/527951

**Published:** 2014-04-29

**Authors:** Anthony Pak-Hin Kong, Jubin Abutalebi, Karen Sze-Yan Lam, Brendan Weekes

**Affiliations:** ^1^Department of Communication Sciences and Disorders, University of Central Florida, HPA-2 106, P.O. Box 162215, Orlando, FL 32816-2215, USA; ^2^Laboratory for Communication Science, University of Hong Kong, Pokfulam, Hong Kong; ^3^Psychology Department, San Raffaele University and San Raffaele Scientific Institute, 20132 Milano, Italy

## Abstract

Neuroimaging studies suggest that the neural network involved in language control may not be specific to bi-/multilingualism but is part of a domain-general executive control system. We report a trilingual case of a Cantonese (L1), English (L2), and Mandarin (L3) speaker, Dr. T, who sustained a brain injury at the age of 77 causing lesions in the left frontal lobe and in the left temporo-parietal areas resulting in fluent aphasia. Dr. T's executive functions were impaired according to a modified version of the Stroop color-word test and the Wisconsin Card Sorting Test performance was characterized by frequent perseveration errors. Dr. T demonstrated pathological language switching and mixing across her three languages. Code switching in Cantonese was more prominent in discourse production than confrontation naming. Our case suggests that voluntary control of spoken word production in trilingual speakers shares neural substrata in the frontobasal ganglia system with domain-general executive control mechanisms. One prediction is that lesions to such a system would give rise to both pathological switching and impairments of executive functions in trilingual speakers.

## 1. Introduction

Aphasia among multilingual speakers is a research topic of increasing importance [1]. Paradis [2] estimated there were at least 45,000 new cases of bilingual aphasia in the United States every year. According to the most recent census report [3], the number of multilingual speakers is expected to grow in the United States. It is therefore reasonable that the number of bilingual speakers with aphasia will increase in the coming years.

One unique feature of multilingual aphasia is involuntary and uncontrolled language switching and mixing [4]. Pathological language switching is characterized by the alternation of utterances from one language to another across sentence boundaries. Pathological language mixing, on the other hand, involves the mixing of elements of two languages in a single utterance [4–7]. Language switching and mixing are considered pathological if they occur involuntarily and are beyond the control of the speaker as in bilingual aphasia [8]. One explanation of these phenomena is that language switching and mixing results from the malfunctioning of a “language control” device that separates the languages of a multilingual speaker during production [9].

Goral et al. [10] described a Hebrew-English-French trilingual speaker with aphasia, EC, who showed a differential pattern of recovery and suggested an asymmetric connection between native language (L1) and nonnative languages. Specifically, EC experienced the least degree of language interference when conversing in L1 (Hebrew), which was the most recovered language, and demonstrated more interlanguage activations when producing narratives in L3 (French), which was the least recovered language. Goral et al. also found that during language production in French, interference from L2 (English) was more frequent than from Hebrew. The authors proposed that interlanguage lexical intrusions observed among multilingual speakers with aphasia could be related to the degree of language similarity (e.g., shared vocabulary) and premorbid pattern of language use in addition to other factors such as the age and manner of language acquisition. Faroqi-Shah and Waked [11] also reported a trilingual speaker with aphasia, NK, who spoke Arabic (L1), French (L2), and English (L3). They reported dissociations between nouns and verbs in which NK demonstrated a pervasive verb production deficit irrespective of the task (confrontation naming and narrative speech) or language of elicitation. Therefore, there were no differential effects of language similarity.

As to the neural locus of the language control device, clinical case studies have shown that damage to a frontal-subcortical circuit not only leads to uncontrolled behavior in brain damaged individuals, but also pathological switching between languages and language mixing [12–14]. Functional neuroimaging studies with unimpaired multilingual speakers have corroborated such findings [15–17] showing that language switching relies on a prefrontal-caudate ACC (anterior cingulate cortex) circuit. However, other findings from neuroimaging studies also suggest that the neural network involved in language control is not specific to bi-/multilingualism but is part of a domain-general executive control system [18, 19]. We report evidence that impaired language control and executive functions are associated with lesions to a partially overlapping cognitive and neural system in a multilingual speaker, Dr. T. This is the first case report of pathological switching [6] that is specifically associated with executive control impairments following damage to the executive control system in the frontal cortex.

## 2. Case Report

**Figure 1:**
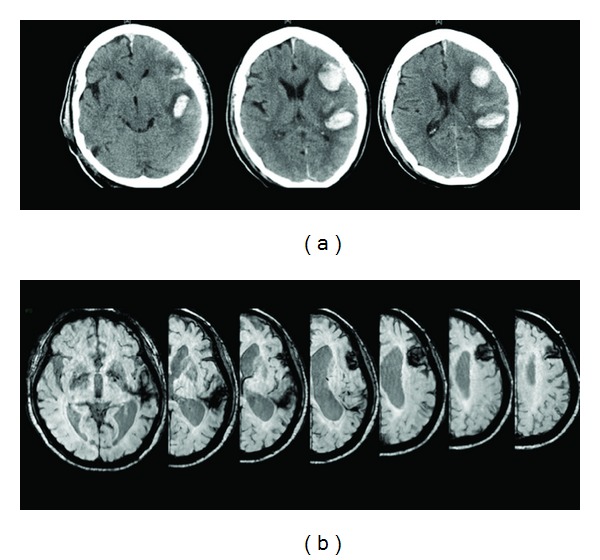
CT scans of the trilingual patient carried out in the acute phase following brain damage reporting the two brain lesions in the left hemisphere (a). MR scanning performed in the chronic phase revealing the extension of the two lesions is illustrated in (b).

**Figure 2:**
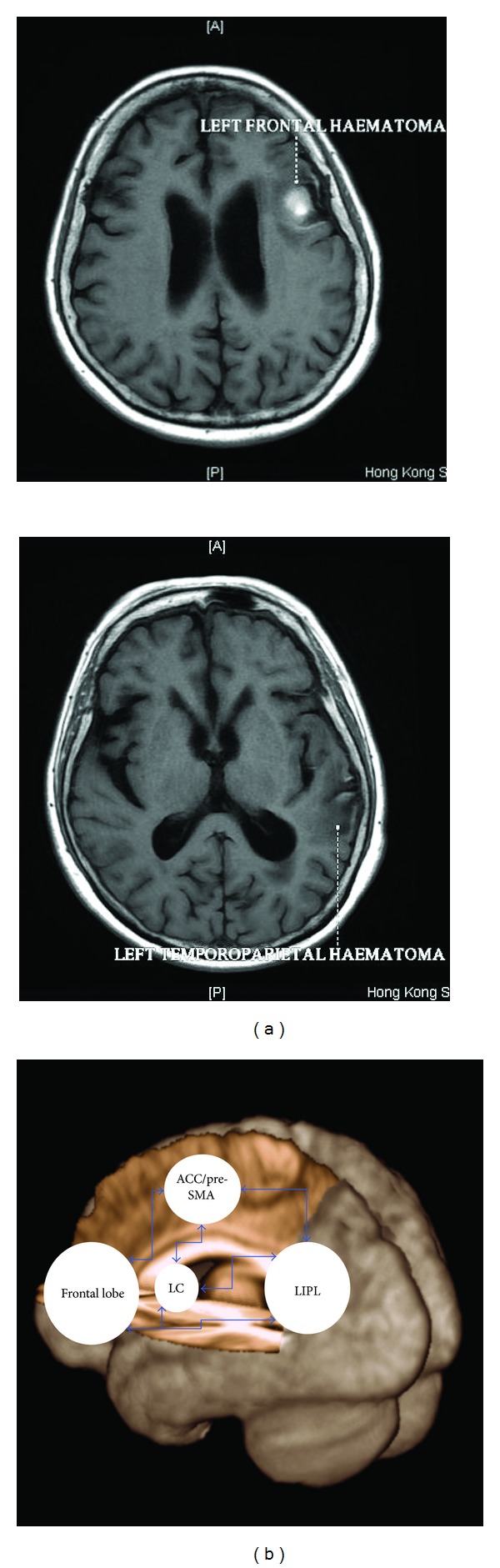
(a) MR scans of the trilingual patient revealing a major haematoma localized in the left frontal lobe and a minor one in the left temporoparietal junction. (b) The neural circuitry involved in language control (adapted from [20]) with the four key areas is identified. The ACC (anterior cingulate cortex) is involved in monitoring functions such as error detection (i.e., if the speaker has selected the correct language), the frontal lobe is involved in error correction and response inhibition, and the left caudate (LC) is involved in supervising the correct selection of the language and language planning while the left inferior parietal lobule (LIPL) along with its right-hemispheric counterpart is involved in more attentional processes such as biasing selection towards and from the language in use. This network resembles the domain-general executive control network (see [20] for details). Of note, the lesions of our trilingual patient reported in (a) may have interrupted the connections between the frontal and parietal areas of this neural circuitry, hence leading to an inability to inhibit the unwanted language (i.e., frontal lobe) and focusing attention on the language in use (i.e., parietal lobe).

Dr. T is a 77-year-old right-handed female trilingual Cantonese-English-Mandarin speaker who sustained a traumatic brain injury causing a fluent aphasia with pathological switching and mixing [8]. CT scanning in the acute phase (Figure 1) and MR imaging in the chronic phase (Figure 2 and bottom row in Figure 1(b)) revealed two lesions, a major one in the left frontal lobe and a minor one in left temporoparietal areas. She was a retired radiologist premorbidly. Her first language, Cantonese (L1), was acquired from birth and used extensively in daily life and at work in Hong Kong. She started to learn English, her second language (L2), formally from the age of 13 years and used English regularly in professional life. Mandarin, the third language (L3), was learned in her early twenties when she obtained her medical degree and worked as a doctor in Mainland China. Premorbidly, Dr. T mainly used Cantonese and English to communicate with her husband in Hong Kong and grandchildren who are living in the United Kingdom, respectively. Dr. T's husband was recruited as a control because he was perfectly matched in age, handedness, education level, and trilingual language knowledge.

Cognitive functions were assessed using Raven's Standard Progressive Matrices [21] and the Symbol Trials of the Cognitive Linguistic Quick Test (CLQT) [22]. According to the smoothed 1986 Raven norms for urban Mainland China [22], the performances of the case and control were within normal limits (50th percentile: 34/60 versus 75th percentile: 46/60). Both participants also scored above the criterion-referenced cut score in the CLQT Symbol Trials (8/10 versus 10/10). These results suggested normal cognitive ability. However, Dr. T scored significantly lower on a modified Stroop color-word test [23] (3/25 versus 25/25) and the Wisconsin Card Sorting Test (WCST) [24] (total error numbers: 2nd versus over 99th percentile; perseverative responses: less than 1st versus 97th percentile; conceptual level responses: 4th versus 99th percentile), revealing impairment of her executive functions.

Based on the Cantonese version of the Western Aphasia Battery (CAB) [25], Dr. T was diagnosed with Wernicke's aphasia in L1, with a total aphasia quotient of 46.6 (out of 100). Specifically, during the spontaneous speech task, Dr. T produced fluent unintelligible jargon and neologisms with severe word retrieval difficulty. She frequently switched between her Cantonese, English, and Mandarin during conversation, which decreased comprehensibility. Auditory comprehension was impaired at the sentence level with difficulty comprehending complex sentences and decontextualized questions as well as following one-step commands. In terms of repetition, Dr. T showed breakdown of performance at two-syllable words. Dr. T's reading and writing abilities were better than verbal comprehension and production. She was able to comprehend written sentences and commands with occasional errors. Reading comprehension ability was significantly better than her reading aloud performance. As for writing ability, Dr. T showed impairment even at the single-word level with better written than verbal naming.

Dr. T's multilingual ability was examined using the Bilingual Aphasia Test (BAT) [26]. Moderate impairment in auditory comprehension and oral production across the three languages was found. Dr. T demonstrated slightly better auditory comprehension abilities in Cantonese (L1: 44%) and Mandarin (L3: 46%) than in English (L2: 39%), but the opposite was observed in oral production (L1: 35%, L2: 46%, and L3: 35%). Dissociations were also observed in her reading comprehension (L1: 75%, L2: 75%, and L3: 85%) and reading aloud (L1: 31%, L2: 73%, and L3: 4%) abilities. To summarize, the BAT revealed Wernicke's aphasia of moderate grade in all three languages for Dr. T, which is consistent with the above-mentioned CAB results. In addition, Dr. T's linguistic profile contrasts with performance over 93% accuracy demonstrated by the control across all BAT tasks on these languages.

Pathological language switching and mixing demonstrated by Dr. T, when compared to the control, were examined in multilingual confrontation naming and discourse production in three languages. Both participants were required to name 85 colored pictures from Snodgrass and Vanderwart [27] in Cantonese, English, and Mandarin. The stimuli were grouped into 18 conversation topics, for which the participants conversed on each topic in three languages on separate days with author K. Lam for at most 15 minutes. The middle six minutes of three selected topics (a subset of the language samples was selected according to the following criteria: (1) the duration of each topic in each language was at least ten minutes, (2) the participants were familiar with the topic in which at least four items overlapped with those in the confrontation naming task, and (3) the maximum amount of neologisms in each topic in each language was less than 25%) were transcribed verbatim and the percentage of correct code-switched words was calculated (the percentage of code switching was calculated based on five parameters adopted in each elicitation, including (1) total number of Cantonese words, (2) total number of English words, (3) total number of Mandarin words, (4) total number of neologisms, and (5) total number of words in all languages including neologisms (i.e., sum of words in parameters one to four). Pauses and intelligible words were used to determine the word boundary for defining neologisms. Each instance of a neologism (regardless of the length) following a pause or an intelligible word was counted as one neologism). Pairwise comparisons revealed significantly less code switching in Cantonese confrontation naming compared to discourse production for the same lexical items (*P* < 0.001). No differences were observed in English (L2; *P* = 0.44) or Mandarin (L3; *P* = 1.00). Chi-square comparisons showed that in confrontation naming, code switching from the target language to Cantonese was significantly more common when targets were given to name in English (L2) and Mandarin (L3). This pattern was generally similar in discourse production, except that more Mandarin words were produced in Cantonese discourse production (L1→L2: 1.1%, L1→L3: 31.0%).  Table 1 displays the code switching pattern of Dr. T. Note that the control, unlike Dr. T, only showed rare-to-absent incidence of code switching behavior. Examples of Dr. T's code switching during confrontation naming and discourse production are given in Tables 2 and 3, respectively. Note that given the high proportion of lengthy unintelligible neologisms produced by Dr. T, which lead to difficulty in determining sentence boundaries, language switching and language mixing could not be differentiated in the present study.

## 3. Discussion

A key question in bilingual language production is the specificity of the language control device that is used by multilingual individuals. Failures in language control may lead to unwanted language switching as observed in some cases of bilingual aphasia [10], and in the case reported here. On the other hand, in healthy subjects, voluntary language switching is considered an instance of task switching as it involves, at a minimum, a switch between different stimulus-response sets.

Based on the results from Goral et al.'s study [10], it could be hypothesized that Dr. T produces more code switching from Mandarin (L3) to Cantonese (L1) because the language pairs are linguistically closer to each other than English (L2) and Cantonese (L1). The data in Table 1 (30.8% and 30.9% code-switched words from L3 to L1 during confrontation naming and discourse production task, resp.) are partly consistent with this hypothesis. On the other hand, the relatively high incidence of switching from English (L2) to Cantonese (L1) by Dr. T, that is, 21.9% and 26.9% code-switched words in confrontation naming and discourse production, respectively, was unexpected. We contend that the pattern of code switching in her language production does not reflect language similarity and is more likely due to the age of acquisition or the language dominance of Cantonese [28, 29].

We found a strong association between pathological language switching and control over task switching on standardized tests of executive control and function. Apart from taking time to invoke new stimulus-response mappings according to a new goal and choosing which attributes to attend to on such tasks, changing tasks might require the inhibition of competing stimulus-response mappings [30]. As such, we contend that language switching engages the same neural network used for task switching, that is, the frontobasal ganglia executive control system circuit (Figure 2). Hence, we would predict that lesions to that system would produce pathological switching and impairments of executive function, such as perseveration errors committed by Dr. T on the WCST.

We believe that Dr. T's pathological code switching can be attributed to impairment in the executive control resulting from damage to the frontal lobe. Interestingly, Dr. T's code switching was significantly less prominent in Cantonese oral confrontation naming compared to Cantonese spontaneous speech. Studies show that code switching can vary depending on the amount of stress in the environment [31]. The increased demand for linguistic, cognitive, and pragmatic skills in connected speech when compared to confrontation naming may pose more cognitive load on the neural system for Dr. T, resulting in limited capacity to regulate her code switching and leading to more frequent code switching in discourse production. The more frequent intrusions of Mandarin words than English words in imposed Cantonese tasks and the more prevalent intrusions of Cantonese words than English words in Mandarin tasks indicate that switches are more likely to the linguistically similar languages (e.g., Cantonese or Mandarin) than the linguistically different language (e.g., English). When English was the target language, a significantly higher proportion of switches were Cantonese than Mandarin, which may be explained by the fact that Cantonese was the dominant language in Dr. T's life.

Our case provides novel empirical evidence about the neural mechanism in bilingual brains. We contend that language control and domain-general executive control are served by a partially overlapping cognitive and neural system. The frontal lobe lesion damaged frontostriatal connections within the control network causing both pathological language switching and impairments to executive function. On the other hand, the lesion in the temporoparietal junction may be responsible for fluent aphasia with no effect upon language and executive control. On the basis of the language and cognitive control model proposed by Abutalebi and Green [8, 9] we cannot rule out the possibility that different lesions may be separately responsible for impaired language control and for impaired executive functions. However, it should be noted that pathological language switching has never been observed after parietal lesions and most typically results from lesions involving the left caudate-frontal lobe circuitry [9]. Left parietal lesions, on the other hand, mostly explain difficulties switching from one language to another, that is, pathological fixation on one language [32]. Likewise, the dysexecutive syndromes reported result from lesions to the frontal lobes [8]. Hence, although we may not totally rule out the possibility that each single lesion was responsible for different deficits (such as the frontal lesion for impaired executive functions and the parietal lesion for impaired language control or vice versa), it is more parsimonious to assume that the frontal lesion was responsible for both impairments. As to the crucial role of the left caudate-frontal lobe circuitry in language control, evidence provided by Mariën et al. [14] shows remission of language mixing and switching is associated with increased perfusion of left frontal lobe and left caudate nucleus. Interestingly, in their bilingual case, perfusional deficits remained in left temporoparietal areas and the patient continued to display fluent aphasia in L1 and in L2. It is of interest that the lesions in the present case were due to head trauma. MR imaging might not be sensitive to microscopic injury or small areas of molecular and/or physiological damage within brain tissues. Therefore, it is possible that the language and executive function deficits demonstrated by Dr. T were at least in part due to additional lesions not seen on the MR imaging. This limits the implications that can be drawn from the present case study.

Recent studies have speculated on the implications of utilizing the same system, in which bilinguals are more proficient in executive tasks than monolinguals [33]. Dr. T, who showed more prominent (higher-incidence and more frequent) switching in connected speech than confrontation naming, may provide insight into the demands for linguistic and cognitive resources in relation to task processing in multilingual speakers.

## Figures and Tables

**Table 1:** Pairwise comparison of code-switched words (%) across naming contexts.

Naming context	Target language	Correct code-switched words (%)			Chi-square
		Cantonese	English	Mandarin	
Confrontation	Cantonese (L1)	—	3.56	6.72	0.82
	English (L2)	21.85	—	4.64	10.70*
	Mandarin (L3)	30.77	1.40	—	28.13**

Discourse production	Cantonese (L1)	—	1.07	30.97	28.13**
	English (L2)	26.93	—	5.65	13.36**
	Mandarin (L3)	30.89	0.52	—	28.13**

Note: *P < 0.01. **P < 0.001.

**Table 2:** Samples of Dr. T's code switching at the single-word level (confrontation naming).

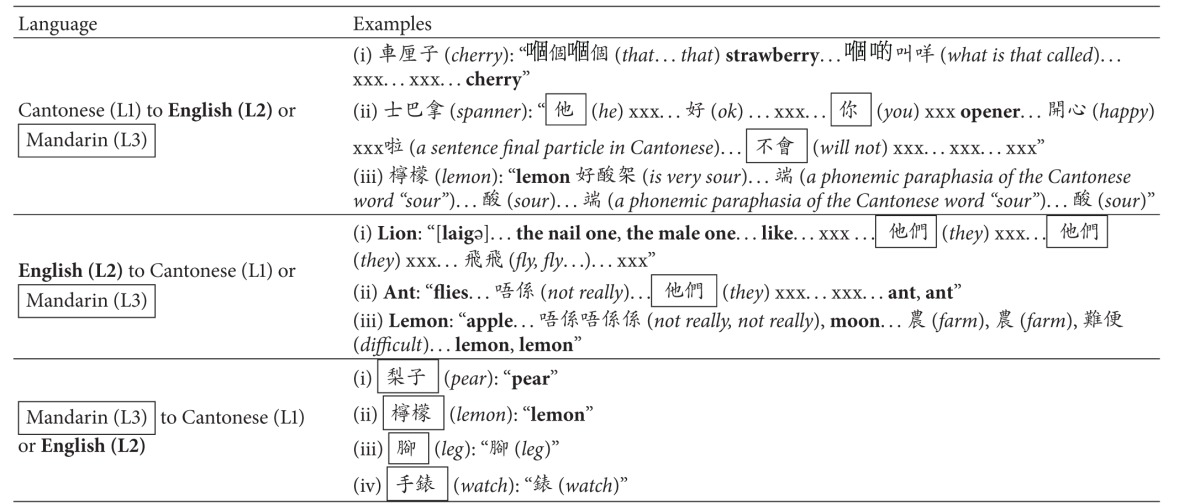

Notes: all verbal responses in English were **bold** and all verbal responses in Mandarin were boxed. Glosses and/or remarks in English were *italicized* and given in parentheses. Unintelligible vocalizations (or jargons) were transcribed as xxx. Note that several xxx strings were used in a row, in case the number of unintelligible words could be distinguished.

**Table 3:** Samples of Dr. T's code switching at the discourse level.

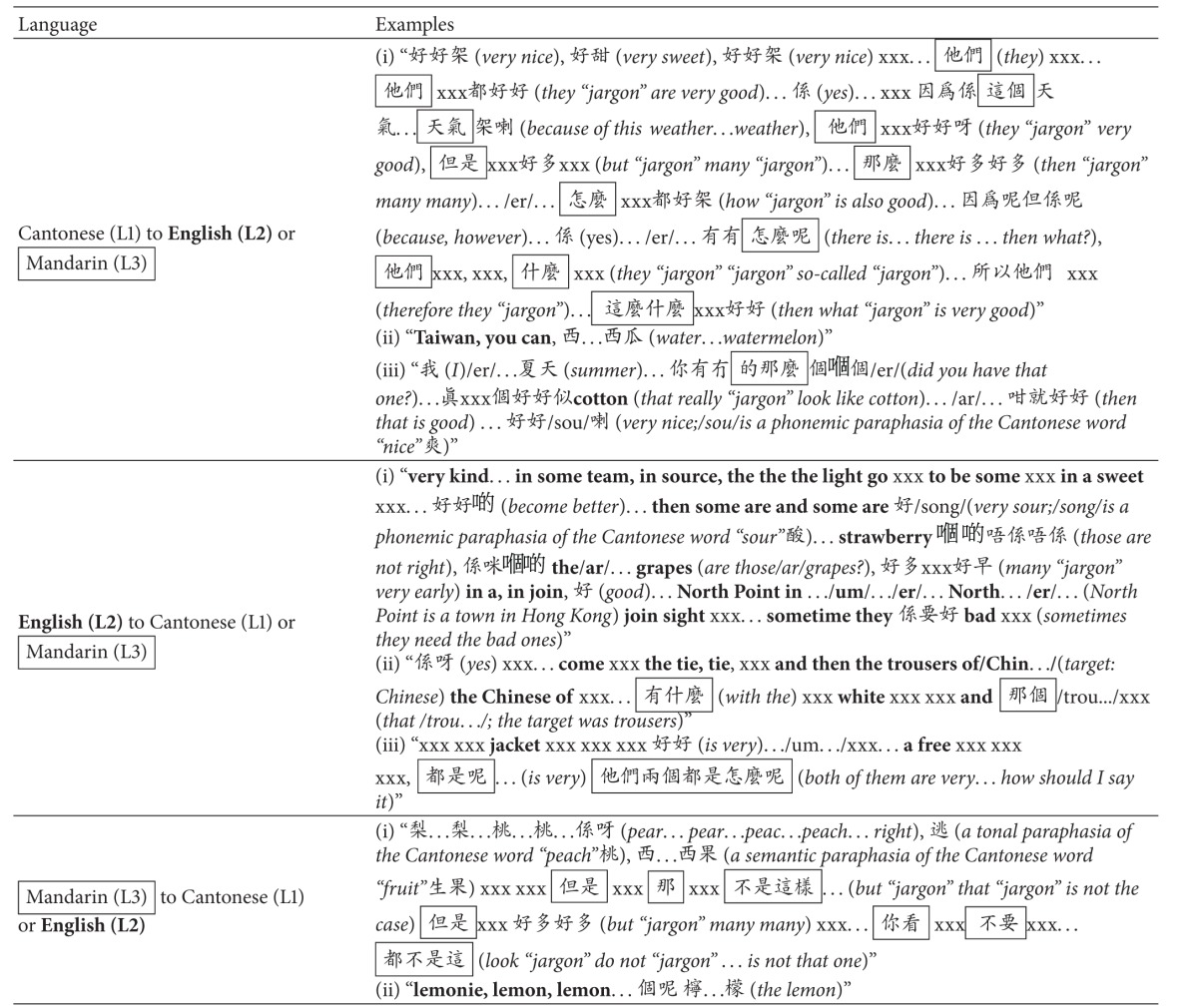

Notes: all verbal responses in English were **bold** and all verbal responses in Mandarin were boxed. Glosses and/or remarks in English were *italicized* and given in parentheses. Unintelligible vocalizations (or jargons) were transcribed as xxx. Note that several xxx strings were used in a row, in case the number of unintelligible words could be distinguished.
